# Supplementary material for: Sterols and Sphingolipids as New Players in Cell Wall Building and Apical Growth of Nicotiana tabacum L. Pollen Tubes
Source: Plants (Basel). 2022 Dec 20;12(1):8. doi: 10.3390/plants12010008 (PMC9824051; doi:10.3390/plants12010008)
Supplement: Supplementary file 1 [file plants-12-00008-s001.zip › plants-2010239-supplementary.pdf]

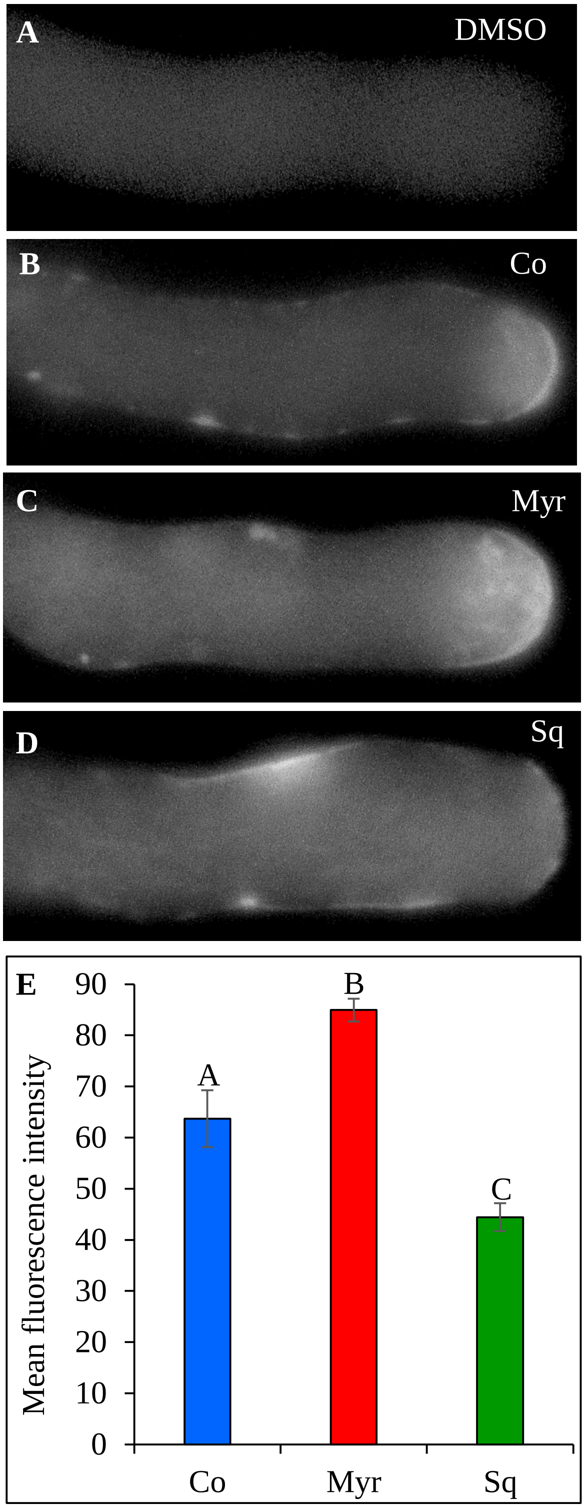

Fig S1

Figure S1. **Filipin staining.**

In LIFEACT-EGFP overexpressed pollen tube, AFs were randomly distributed and short bundles were observed in the shank and tip of pollen tubes. (**A**; arrows). Bright field image of the pollen tube in A was showed (**B**). Magnification Bar = 10 µm.

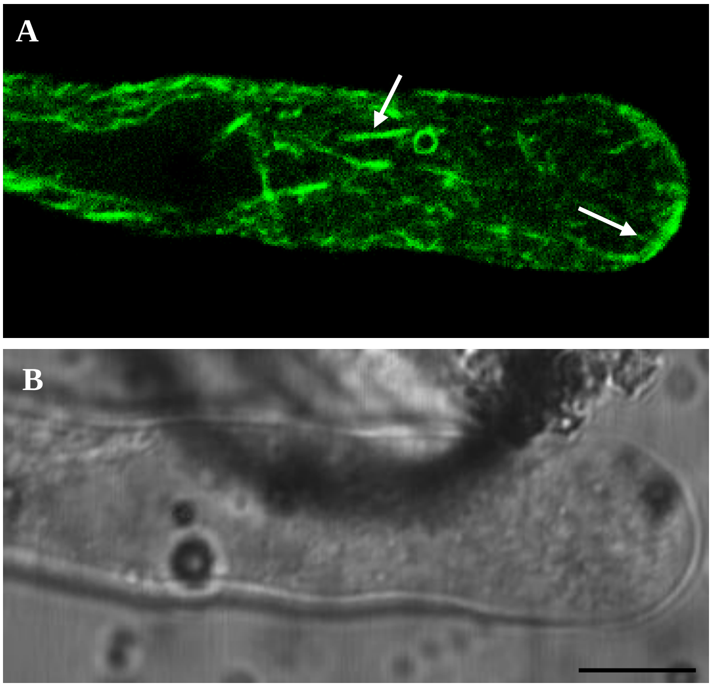

Figure S2

Figure S2. pLat52-LIFEACT-EGFP overexpressing pollen tubes. In LIFEACT-EGFP overexpressed pollen tube, AFs were randomly distributed and short bundles were observed in the shank and tip of pollen tubes. ((**A**); arrows). Bright field image of the pollen tube in (**A**) was showed (**B**). Scale Bar = 10 μm.

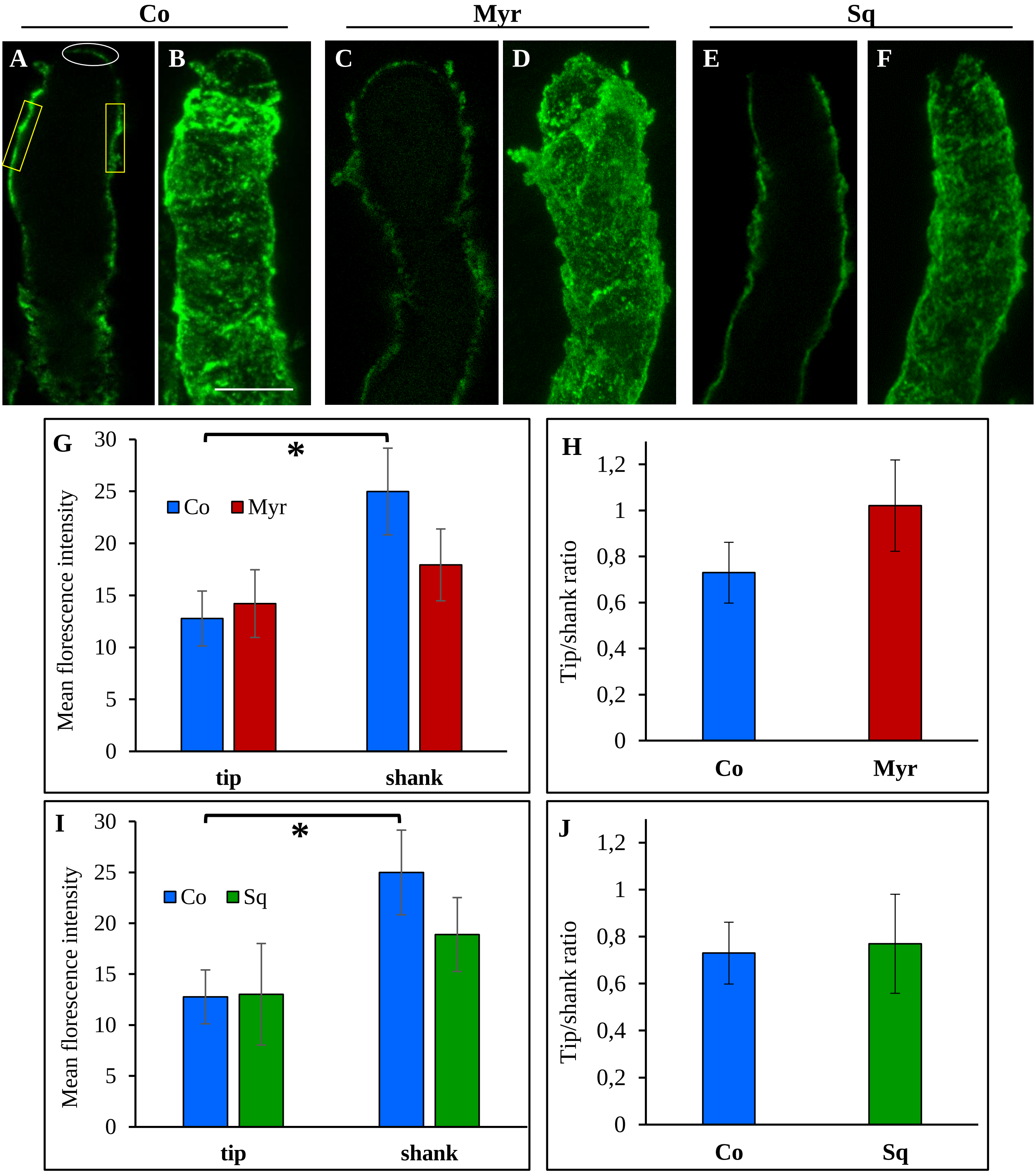

Fig S3

Figure S3. Effect of squalestatin on LEP distribution. Distribution of low-esterified pectins was determined by labelling with LM19 antibody in control (**A**,**B**), myriocin- (**C**,**D**) and squalestatin- (**E**,**F**) treated pollen tubes. Medial plane (**A**,**C**,**E**) and whole reconstruction (**B**,**D**,**F**). Quantification of fluorescence intensity in tip and shank (white and yellow ROIs, respectively, as specified in panel (**A**); n > 12) using ImageJ showed that no significant differences with respect to control were induced by myriocin and squalestatin treatment of pollen tubes (**G**,**I**); ANOVA and Tukey's post hoc test: $p > 0.05$, reported as *). The difference in esterified pectins between tip and shank is maintained in pollen tubes grown in the presence of inhibitors (**H**,**J**); ANOVA and Tukey's post hoc test: $p > 0.01$). Scale Bar = 10 µm.

Supplementary Materials and Methods

Data analysis of tube growth dynamics

To measure tip position and wall thickness we used a custom-written Matlab analysis pipeline.

The data is a time-lapse video of pollen tube growth. To identify tip position in each frame, we first extract an overall segmentation mask combining information about the max projection, the SD of the max projection and the SD over time. To extract the mask, an overall threshold T is applied.

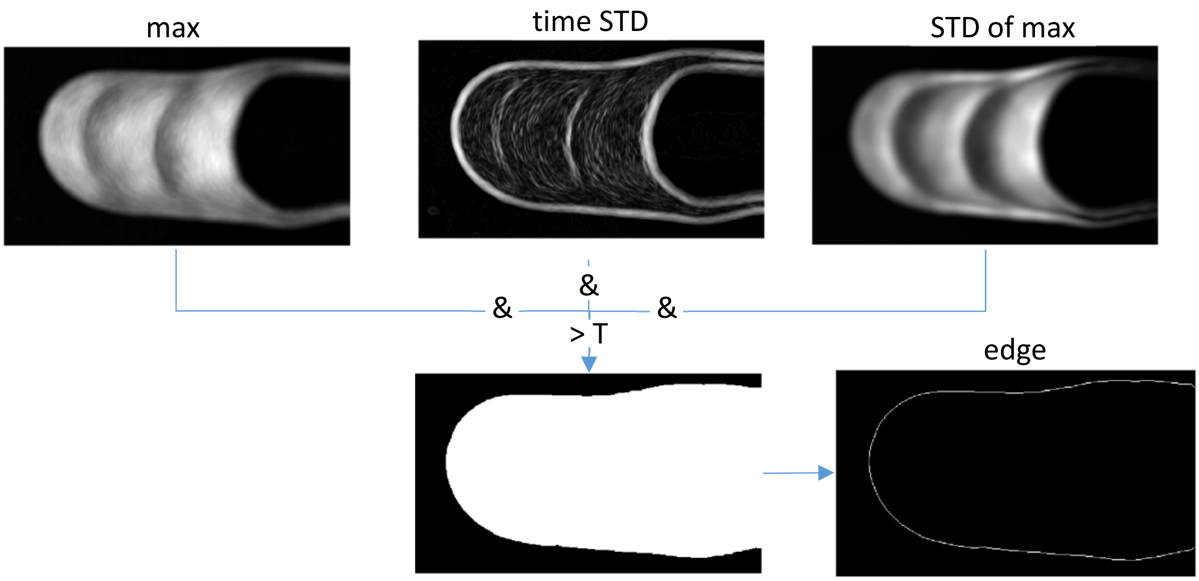

Then we identify the edge and build an oriented trail from one end to the other. To do so we identify the first and last point of the trail from the number of first neighbours of the contour; only the extreme points have a single nearest neighbour. We find the central line by averaging the coordinates of opposite pairs of points, like the first and last point, the second and second last, and so on (points with the same colour in the image below). This central line follows the growth path and its intersection with the tube in each frame of the time lapse movie provides a reference for more accurate localization of tip position. This first step of growth path detection enables us to include any tube with any curvature in the analysis, the only condition being that it always remains in the focal plane.

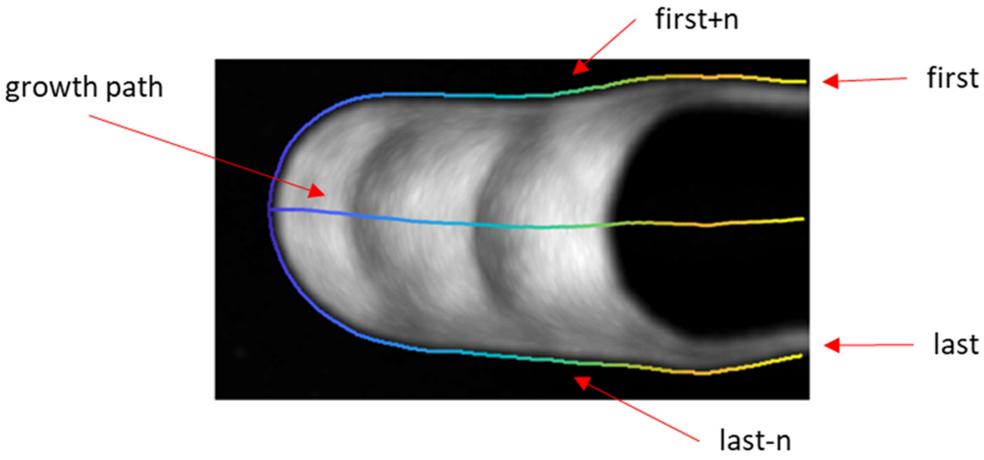

The next step is precise localization of tip position and determination of wall thickness. We segment the profile of the tube in each frame, then we identify the intersection between the tube mask and the growth path. The pixels that correspond to the intersection are approximated with a straight segment and the values of the corresponding pixels are considered in analysis of the intensity profile.

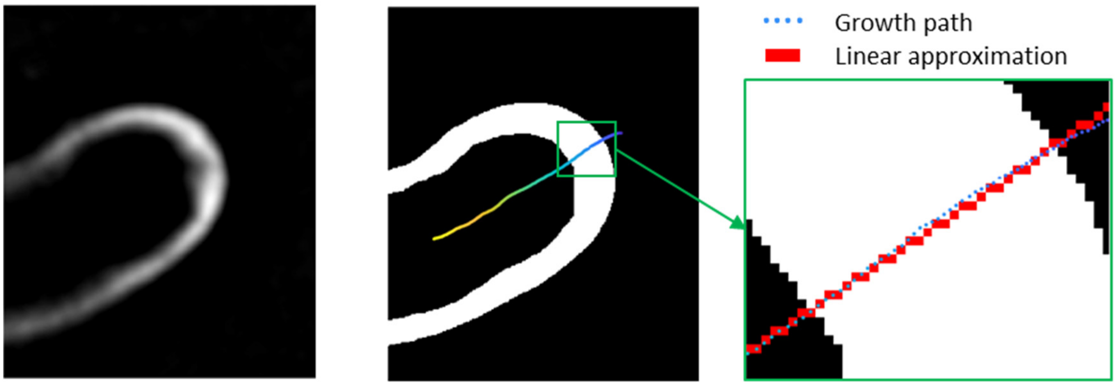

Three parallel profiles are averaged to reduce noise. The profile is fitted to a Gaussian curve; from the fit we get the position of the tube tip and wall thickness as the max position and standard deviation of the Gaussian curve.

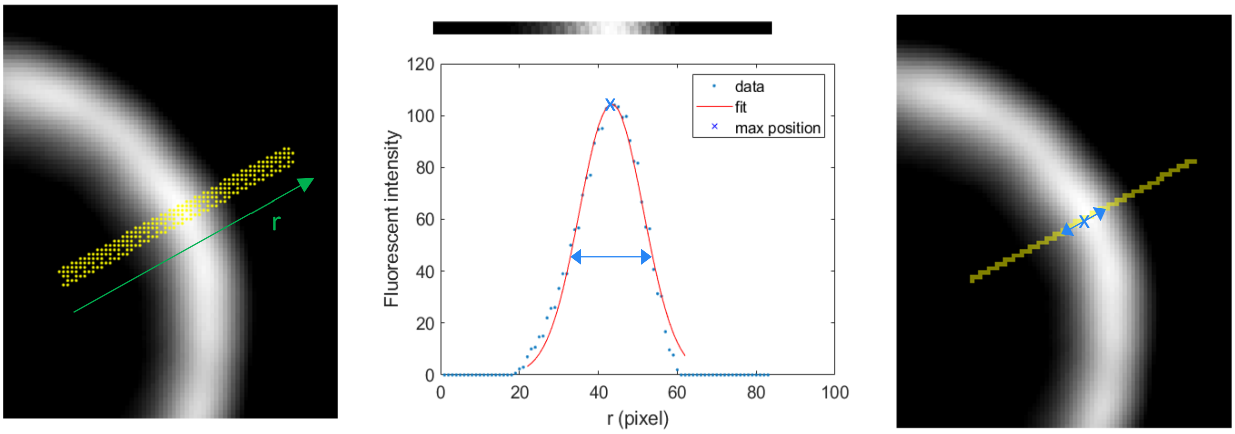

Note that the value of the parametric coordinate across the tube wall (r in the figures) is not the number of pixels of the intensity profile but is adjusted according to the slope of the curve, rather than the pixellation of the image. Indeed, the distance between the centre of consecutive pixels touching just by the corner is √2 times the distance between pixels disposed side by side.

Statistical analysis of pectin methyl esterase, callose and cellulose distribution

Comparison of fluorescence in the tips and shanks of pollen tubes treated with different substances, was performed with mixed models assuming a Gaussian error distribution. Values were first averaged within the tube region to obtain two values per tube, one for the tip (distance from apex <5 µm) and one for the shank (distance from apex 5-15 µm). Predictors were the treatment (three-level factor) and the region (two-level factor). Their interaction was included in the aniline blue analysis, but removed from that of CBM3A because it was far from significant. Models also included tube identity as a random factor and region as a random covariate of tube identity. With this parameterization, the mixed model performed pairwise comparison of the values of the two regions in a pollen tube while accounting for the treatment effect. These models were fitted using the LME procedure of the NLME package of R 4.0.5 (R Core Teams 2021). The R codes used for the analyses were:

lme(fluorescence.values ~ Treatment+Region random = ~ 1 + Region|TubeID, data = CBM3A)

lme(fluorescence.values ~ Treatment*Region, random = ~ 1 + Region|TubeID, data = Aniline)

These analyses were followed by post-hoc tests whereby we compared the treatment groups with the control (Dunnett's method) using estimated marginal means, pairs and contrast procedures of the emmeans package. For detailed results of the models, see Tables S1 (CBM3A) and S2 (Aniline blue).

Table S1: Results of the mixed model of variation in mean fluorescence in tubes treated with CBM3A

**Effect**	**Coef**	**SE**	**DF**	**t-value**	**p-value**
Intercept	29.496	1.148	74	25.696	<0.001
Myriocin	2.440	1.606	74	1.519	0.133
Squalestatin	-2.755	1.655	74	-1.665	0.100
Shank	11.243	1.093	74	10.290	<0.001

Table S2: Results of the mixed model of variation in mean fluorescence in tubes treated with aniline blue

**Effect**	**Coef**	**SE**	**DF**	**t-value**	**p-value**
Intercept	8.509	2.352	67	3.618	<0.001
Myriocin	-5.470	3.358	67	-1.629	0.108
Squalestatin	7.282	3.784	67	1.924	0.059
Shank	28.312	5.029	65	5.630	<0.001
Shank* Myriocin	-7.141	7.182	65	-0.994	0.324
Shank* Squalestatin	26.998	8.013	65	3.369	0.001

Analysis of variation of fluorescence along the pollen tube margins was performed with non-linear mixed models (NLMMs), which are very flexible statistical tools that can model any parameter of growth curves as a function of different predictors. This flexibility also extends to the random part of the model, because it is possible to enter different random structures for each parameter of the growth curve. However, fitting NLMMs is challenging. To reduce the complexity of these models, we ran preliminary analyses to assess: 1) which growth curve fit the growth trajectory of each morphological trait best; 2) which parameters of the growth curves showed large variability between tubes, so as to properly parameterize the random part of the NLMMs. The interpolated functions were:

- Gaussian function

$$y = A + Be^{-\frac{1}{2}\left(\frac{x-\mu}{\sigma}\right)^2}$$

where x is the distance, A is the height of the tails of the curve, B is proportional to the height of the peak, e is the base of the natural logarithm, μ is the distance of the peak, and σ is the width of the peak.

- Three-parameter logistic function

$$y = \frac{K}{1 + e^{\frac{i-x}{s}}}$$

where s is the scale parameter that indicates the maximum growth rate of the curve (curves with larger s-values are flatter to the left and increase more steeply), K is the upper asymptote, i is the inflection point (i.e. the distance at which growth is fastest), and e is the base of the natural logarithm.

- Four-parameter logistic function

$$y = L + \frac{K - L}{1 + e^{\frac{i-x}{s}}}$$

where L is the lower asymptote of the curve (i.e. initial value) and the other parameters are as above.

To assess the structure of the random part of the NLMM, we used the procedure described in Morganti et al. (2017). First we interpolated the selected growth curves with the data of individual plants. Then we plotted the ranges of the parameters from curves fitted to individual plants and noted heterogeneous ones by visual inspection (see Sicurella et al., 2014 and Morganti et al., 2017 for a similar approach). Finally, trait variance also usually increases with size. In the final NLMMs, we therefore assumed: a) random variation of parameters showing wide heterogeneity, b) variation of the variance with distance (CBM3A and aniline blue) or with the fitted value (pectin methyl esterase) according to an exponential function, as suggested in Oswald et al. (2012). In the fixed part of the model, we initially allowed for variation of all model parameters between treatments, but then removed non-significant effects for the sake of simplicity.

The models were fitted by the NLME procedure of the NLME package of R 4.0.5. Tables S3, S4 and S5 show the detailed results of the models.

Table S3: Results of the non-linear mixed model of variation in fluorescence in tubes treated with pectin methyl esterase. The fitted function is the Gaussian curve.

**Effect**	**Coef**	**SE**	**DF**	**t-value**	**p-value**
μ	19.879	0.415	17662	47.949	<0.001
σ	3.631	0.080	17662	45.134	<0.001
σ* Myriocin	-0.245	0.119	17662	-2.067	0.039
σ* Squalestatin	-0.564	0.102	17662	-5.554	<0.001
B	111.135	1.922	17662	57.837	<0.001
B * Myriocin	8.756	3.088	17662	2.835	0.005
B * Squalestatin	60.212	3.379	17662	17.819	<0.001
A	128.454	0.933	17662	137.609	<0.001
A* Myriocin	-18.463	1.491	17662	-12.381	<0.001
A* Squalestatin	42.904	1.456	17662	29.463	<0.001

Table S4: Results of the non-linear mixed model of variation in fluorescence in tubes treated with CBM3A. The fitted function is the four-parameter logistic curve.

Effect	Coef	SE	DF	t-value	p-value
L	30.398	0.672	18735	45.254	<0.001
K	224.722	18.972	18735	11.845	<0.001
K* Myriocin	-64.709	26.027	18735	-2.486	0.013
K* Squalestatin	-42.345	26.53	18735	-1.596	0.111
i	16.725	0.297	18735	56.288	<0.001
s	1.088	0.022	18735	49.859	<0.001
s* Myriocin	0.145	0.035	18735	4.143	<0.001
s* Squalestatin	-0.201	0.03	18735	-6.648	<0.001

Table S5: Results of the non-linear mixed model of variation in fluorescence in tubes treated with aniline blue. The fitted function is the three-parameter logistic curve.

Effect	Coef	SE	DF	t-value	p-value
K	503.629	7.828	16990	64.34	<0.001
K* Myriocin	-166.281	8.099	16990	-20.53	<0.001
K* Squalestatin	-116.097	9.095	16990	-12.765	<0.001
i	28.394	1.764	16990	16.094	<0.001
i* Myriocin	-3.99	2.502	16990	-1.595	0.111
i* Squalestatin	-7.329	2.79	16990	-2.626	0.009
s	5.938	0.424	16990	14.02	<0.001
s* Myriocin	-1.751	0.602	16990	-2.911	0.004
s* Squalestatin	-0.811	0.670	16990	-1.21	0.226
